# Social, economic, and physical side effects impact PrEP uptake and persistence among transgender women in Peru

**DOI:** 10.1186/s12889-024-19474-x

**Published:** 2024-07-25

**Authors:** Sarah Naz-McLean, Jesse Clark, Leyla Huerta, Kenneth H. Mayer, Javier R. Lama, Sari Reisner, Amaya Perez-Brumer

**Affiliations:** 1 Department of Epidemiology, Dalla Lana School of Public Health, University of Toronto, Health Sciences Building 155 College Street, Toronto, ON M5T 3M7 Canada; 2 Department of Medicine, Division of Infectious Diseases, Brigham and Women’s Hospital, Boston, MA USA; 3 Department of Medicine, Division of Infectious Diseases, University of California Los Angeles David Geffen School of Medicine, Los Angeles, CA USA; 4 Féminas, Lima, Peru; 5 The Fenway Institute, Fenway Health, Boston, MA USA; 6 Division of Endocrinology, Diabetes, and Hypertension, Brigham and Women’s Hospital, Boston, MA USA; 7 Asociacion Civil Impacta Salud y Educacion, Lima, Peru; 8 Department of Epidemiology, Harvard T.H. Chan School of Public Health, Boston, MA USA; 9 Division of Social and Behavioural Health, Dalla Lana School of Public Health, University of Toronto, Toronto, Canada; 10 Department of Epidemiology, University of Michigan School of Public Health, Ann Arbor, USA

**Keywords:** PrEP, HIV, Transgender women, Peru

## Abstract

**Introduction:**

Oral pre-exposure prophylaxis (PrEP) for HIV-1 infection is over 99% effective in protecting against HIV acquisition when used consistently and appropriately. However, PrEP uptake and persistent use remains suboptimal, with a substantial gap in utilization among key populations who could most benefit from PrEP. In Latin America specifically, there is poor understanding of barriers to PrEP uptake and persistence among transgender (trans) women.

**Methods:**

In April-May 2018, we conducted qualitative interviews lasting 25–45 min as part of an end-of-project evaluation of TransPrEP, a pilot RCT that examined the impact of a social network-based peer support intervention on PrEP adherence among trans women in Lima, Peru. Participants in the qualitative evaluation, all adult trans women, included individuals who either (1) screened eligible to participate in the TransPrEP pilot, but opted not to enroll (*n* = 8), (2) enrolled, but later withdrew (*n* = 6), (3) were still actively enrolled at the time of interview and/or successfully completed the study (*n* = 16), or (4) were study staff (*n* = 4). Interviews were audio recorded and transcribed verbatim. Codebook development followed an immersion/crystallization approach, and coding was completed using Dedoose.

**Results:**

Evaluation participants had a mean age of 28.2 years (range 19–47). When describing experiences taking PrEP, participant narratives highlighted side effects that spanned three domains: physical side effects, such as prolonged symptoms of gastrointestinal distress or somnolence; economic challenges, including lost income due to inability to work; and social concerns, including interpersonal conflicts due to HIV-related stigma. Participants described PrEP use within a broader context of social and economic marginalization, with a focus on daily survival, and how PrEP side effects negatively contributed to these stressors. Persistence was, in some cases, supported through the intervention’s educational workshops.

**Conclusion:**

This research highlights the ways that physical, economic, and social side effects of PrEP can impact acceptability and persistence among trans women in Peru, amplifying and layering onto existing stressors including economic precarity. Understanding the unique experiences of trans women taking PrEP is crucial to informing tailored interventions to improve uptake and persistence.

**Supplementary Information:**

The online version contains supplementary material available at 10.1186/s12889-024-19474-x.

## Introduction

Oral pre-exposure prophylaxis (PrEP) for HIV-1 infection is a therapeutic prevention technology that is over 99% effective in protecting against HIV acquisition when taken consistently [1]. In areas with the highest PrEP use, PrEP and treatment as prevention have been evidenced to be highly effective at reducing incident HIV infections on a population level [2–4]. However, PrEP uptake and persistent use are suboptimal among certain key populations who could most benefit from PrEP, such as transgender (trans) women, among whom the global pooled prevalence of HIV infection is 19.9% [3, 5, 6]. Importantly, this poses a prevention gap to reaching UNAIDS 2030 targets to end the AIDS epidemic, as rates of new infections are not declining in settings where HIV is concentrated among key populations [5]. Illustrating this trend, the HIV epidemic in Latin America is concentrated, with 54% of incident cases diagnosed among MSM and trans women. Despite expansion of HIV services, the region experienced a 5% increase in new infections between 2010 and 2021 [7].

In Peru, as in the larger Andean region of Latin America, the HIV epidemic is concentrated in the key populations of MSM and trans women. While the HIV prevalence in Peru’s general population is 0.4%, among trans women the prevalence is estimated to be over 30% [8]. A high proportion of Peruvian trans women are likely to meet the indication criteria for PrEP (for example, 64% report sex work as a primary means of income) [9]. Scale-up of PrEP in Peru could be impactful; a recent modelling study demonstrated the cost-effectiveness of PrEP as a prevention strategy, finding that a combination of interventions including PrEP may avert up to 50% of new infections among Peruvian trans women sex workers and their stable clients over the next 10 years [10]. Yet, PrEP rollout in Peru has been slow. While Truvada™ was approved for use as PrEP by the Peruvian Ministry of Health in 2016, generic oral PrEP has only recently been made available in Peru. Delayed inclusion in the national health system and expensive out-of-pocket cost for private use have resulted in limited availability outside of demonstration projects [11]. In 2023, Peruvian Ministry of Health guidelines were updated to include daily and event-driven formulations of oral PrEP for priority populations; newer long-acting injectable regimens were not included [11–13].As of July 2024, fewer than 3000 people are estimated to be taking PrEP in Peru [14]. While there are limited demographic data available on PrEP users outside of research contexts, long-term retention and adherence among trans women in research studies has been low. According to findings from the TransPrEP study, nearly one-third of participants were lost to follow-up immediately after enrollment and never returned for their first PrEP refill at the one-month mark [15]. After three months, fewer than half of the participants were retained in the study. Of those retained in the study, tenofovir levels in hair samples suggested that less than a third of the intervention arm and less than 10% of the control arm had taken 4 or more doses per week (indicating sufficient protection). A more recent multi-country study, the ImPrEP demonstration project, found similar results, with only 44.5% of Peruvian trans women in the sample remaining engaged with PrEP care after one year [16].

Unfortunately, this finding is not unique to Peru and has been mirrored in numerous global settings, where levels of PrEP uptake among trans women remain low, and PrEP discontinuation high [3, 17–19]. In one US-based sample of HIV-negative trans women, 92% were aware of PrEP, but only 32% had used it in the past 12 months [20]. Recent work has sought to elucidate barriers to PrEP uptake and long-term persistence (defined as “sustained use” over at least 6 months, or during a period of continued risk for acquiring HIV) among trans women. Much of this literature has revealed that experienced and anticipated side effects are important barriers to PrEP uptake and continued use [3, 21–23].

Common physical side effects of PrEP include nausea, flatulence, diarrhea, headache, and abdominal pain [24]. In the iPrEx open-label extension (OLE) trial, which included MSM and trans gender women, these symptoms were described as typically presenting as a “start-up syndrome” lasting as long as one month for some, but usually remitting over time [24]. Additional anticipated side effects that play a role in acceptability among trans women include fears of drug interactions decreasing the effectiveness of gender-affirming hormone therapy [21, 22], even though the literature does not suggest that antiretrovirals affect hormone concentrations [25–27].

Beyond physical side effects, recent work with trans communities has begun to disentangle important barriers across multiple domains of health and well-being [3, 21, 22]. Economic barriers to PrEP uptake include the cost of medication in a setting of extensive social and economic exclusion (i.e., food or housing precarity) and structural barriers to healthcare access, such as time and transportation [3, 21, 23, 28, 29]. Further, PrEP is associated with stigma of being perceived as “at risk” for HIV or living with HIV [3, 21, 23]. Finally, transphobia in healthcare settings may contribute to mistrust of PrEP and less willingness to engage with PrEP services [3, 21, 23, 29, 30]. Among the few studies that have examined PrEP persistence among trans women, findings indicate that barriers to continued use may be similar to those for PrEP initiation, but also include factors like changes in individuals’ risk perceptions over time (e.g., no longer being sexually active) [3, 17]. Notably, much of this work has been conducted in US settings and research is needed to better understand dynamics that limit PrEP uptake and persistent use in low and middle-income country (LMIC) settings.

In Peru, trans women face widespread barriers to accessing HIV services, and discrimination within healthcare settings is common [31, 32]. While barriers to HIV care are well-documented, there is a paucity of research on PrEP experiences in this setting, how these experiences interact with existing societal oppressions, and how these factors impact uptake and persistence. Understanding the experiences of Peruvian trans women who take PrEP, and those who are offered PrEP at no cost but decline, is critical to informing PrEP research and implementation science that is responsive to the needs of trans women. Calls in the literature have highlighted the need for more nuanced, qualitative investigations of factors that influence PrEP uptake and persistence among trans women, particularly in global south settings [16, 17]. Given the importance and potential impact of addressing this gap in Peru, this study describes trans women’s experiences of PrEP use, including the physical, social, and economic side effects PrEP, and how these experiences impact PrEP acceptability, uptake, and persistence.

## Methods

This manuscript presents findings from a cross-sectional qualitative end-of-study evaluation of the TransPrEP study (R34 MH105272; clinicaltrials.gov registration #NCT02710032, trial registration date 16/03/2016). The full study methodology has been previously reported [15]. Briefly, between September 2017 and July 2018, a cluster randomized controlled trial was implemented to evaluate the impact of social network-based peer support on longitudinal PrEP adherence among trans women in Lima, Peru. Recruitment followed a limited-chain referral approach to form clusters based on existing social networks within distinct geographic areas of Lima. Trans women recruited underwent individual eligibility screening followed by two counseling sessions to learn about PrEP, including the potential risks and benefits, prior to deciding whether to begin PrEP use. Participants who were eligible (HIV-negative adult trans women reporting condomless anal sex in the prior 6 months) and interested in beginning PrEP could then opt-in to enroll in the study and collect their first bottle of PrEP medication. Participants were randomized in clusters to either control or intervention arms after enrollment. All participants received six months of PrEP treatment, regular HIV and safety lab testing, and longitudinal follow-up surveys. Clusters randomized to the intervention arm additionally received structured adherence support for PrEP through bi-weekly peer-led workshops.

### Sample and recruitment

Data presented here are derived from an end-of-project qualitative evaluation conducted between April-May 2018. All participants previously consented to be re-contacted, even if they chose not to enroll in the TransPrEP study. Individuals were eligible to be included in the evaluation if they: (1) screened and were eligible to participate in the TransPrEP study, but opted not to enroll, (2) enrolled in the study but later withdrew, (3) were actively enrolled at the time of interview and/or had recently completed the study, and (4) were study facilitators for peer workshops. As a community-informed study, study facilitators were trans women with experience in peer navigation, advocacy and community leadership, and research coordination. Individuals were considered ineligible to join the qualitative end-of-project evaluation if they did not consent to be re-contacted by the study team, if they could not be reached using contact information on file, or if they did not meet the baseline eligibility criteria to enroll in the TransPrEP study (for example, trans women living with HIV).

We aimed to recruit approximately equal numbers of participants who were actively enrolled or had discontinued or refused participation. Participants were recruited by the lead facilitator, who purposively sampled participants from each category to gain a range of experiences (e.g., intervention vs. control arm, geographical area of Lima, and enrollment status). The study facilitator contacted potential participants by email, phone call, or text message (based on the participant’s preferred contact method) to invite them to participate in a one-time qualitative interview.

### Data collection procedures

Following written informed consent, interviews were conducted in Spanish by one primary interviewer (APB), while a secondary interviewer (SNM) observed and made notes of key themes and interview dynamics. Interviews lasted approximately 20–45 min in duration and participants were compensated 15 soles (approximately 5 USD) for their time. Interviews were audio recorded and transcribed verbatim. For de-identification purposes, transcripts were identified only by randomly assigned pseudonyms that were used in the analysis and presentation of findings below.

### Qualitative domains

Interviews followed a semi-structured guide (see Additional File 1) including the following domains: perceptions of the TransPrEP study, participation in biomedical HIV research generally, experiences of taking PrEP (including side effects and PrEP-related stigma), and acceptability of PrEP as an HIV prevention tool for trans women in Peru.

### Data analysis

Codebook development followed an immersion/crystallization approach to identify themes and relationships between themes [33]. Immersion–Crystallization is an inductive, iterative method used throughout the data collection, analysis, and representation phase [34]. Throughout data collection, the primary, secondary interviewer, and lead trans facilitator met daily to discuss notes, reflect on each interview, discuss emergent themes, and refine the question guide. Following data collection, two independent reviewers conducted the coding [SNM and APB], and a third study team member [JLC] resolved any discrepancies. Combining inductive and deductive methods, this analytic approach builds on the semi-structured interview guide, which covered domains listed above, through an iterative process to review transcripts and collaboratively develop a codebook. SNM and APB reviewed transcripts independently to identify themes, which were then discussed to refine. Initial themes were then iteratively discussed with a larger team, including trans study facilitators, over a series of meetings to create a codebook. New patterns, themes, and insights identified during coding were documented with qualitative memos and discussed as a group. The coding was conducted in Spanish to retain linguistic nuance, with quotes translated to English for final publication.

### Ethics

The UCLA Office for Human Research Participant Protections (IRB #13-001898) and the Comite de Etica of the Asociacion Civil Impacta Salud y Educacion (IRB #0089-2014-CE) approved the protocols and study materials for the qualitative evaluation.

## Results

Interviewees (*n* = 34, see Table 1) (mean age 28.2 years, range 19–47) included participants who were actively enrolled in the study (*n* = 16, 47%), participants who enrolled but dropped out (*n* = 6, 18%), participants who screened for the study and were eligible, but opted not to enroll (*n* = 8, 24%), and study facilitators (*n* = 4, 12%). The most frequently reported occupation was sex worker (*n* = 16, 47%) followed by sex worker plus supplementary employment in retail, bars, or as a hair stylist (*n* = 6, 18%) and study coordinator or health promoter (*n* = 4, 12%).

**Table 1:** Sample demographics and enrollment status of participants in the qualitative end-of-project evaluation (*n* = 34)

Variable	
	**Mean (Range)**
Age	28 (19–47)
Enrollment Status	**N (%)**
Facilitators	4 (12%)
Screened, Never Enrolled	8 (24%)
Enrolled, Dropped Out	6 (18%)
Enrolled, Ongoing	16 (47%)
Occupation	
Sex Worker	16 (47%)
Sex Work & Additional Jobs	6 (18%)
Health Promoter, Study Coordinator	4 (12%)
Hair Stylist	3 (9%)
Retail Sales	2 (6%)
Other	3 (9%)
Length of Time on PrEP	
*Actively Enrolled & Taking PrEP at Time of Interview*	
On PrEP 1–2 months	1 (3%)
On PrEP for 2–3 months	5 (15%)
On PrEP for 5–6 months	10 (29%)
*Discontinued PrEP & Dropped Out of Study*	
Used PrEP 1 Month or Less	4 (12%)
Used PrEP Intermittently or on an Ad Hoc basis	2 (6%)
*Never initiated PrEP*	
Participants (Declined to Enroll)	8 (24%)
Facilitators	4 (12%)

Table 1 Legend: Table 1 describes participants who were included in an end-of-project qualitative evaluation of the TransPrEP Pilot RCT.

### Physical side effects

Participants described a range of physical side effects that they attributed to PrEP, ranging from little or no side effects to severe nausea, vomiting, diarrhea, bloating, gas, fatigue, and headache. “It made me sleepy, sleepy, more sleepy. When I took this, I would fall asleep.” (Maribel, age 24, on PrEP for one month, withdrew from study) In some cases, participants described feeling exhausted, dazed, or out of sorts: “at the beginning the first two weeks, three weeks, yes, I felt… it was also that I felt drugged.” (Yessica, age 27, on PrEP six months, actively enrolled).

Some participants noted that physical side effects resolved within one to three weeks of PrEP initiation and did not impact their willingness to continue: “This affected me like just a few days, from then until now, everything’s fine.” (Victoria, Age 28, on PrEP six months, actively enrolled) Similarly, another participant described the side effects of the first several days and noted the importance of taking PrEP with food to alleviate her symptoms. However, she had planned to discontinue PrEP if side effects lasted longer than one week:

“First, when I took it, it made me sick, the first day for about three days I had a headache, my stomach ached, chills, and I was like… I was scared too… I recovered, I kept taking the pill, I thought, I told myself if I’m like this in a week, I won’t take it anymore, but in three days it left me, every lunchtime I take it because if I take it on an empty stomach it shocks me.*”* (Esmeralda, Age 31, on PrEP, actively enrolled).

Others described persistent long-term side effects lasting months, however, this was not always linked to study discontinuation. For example, one participant in the final month of the study who had taken the medication for nearly six months commented, “In my case, I always have a little discomfort here on my head and a little looseness in my stomach, now, since I started taking the pill.” (Verónica, age 47, on PrEP five months, actively enrolled).

Ines described that while some initial side effects (dizziness) resolved, she continued to experience ongoing gastrointestinal discomfort at the time of interview, “My body already assimilated, but it’s just also the discomfort, always when I take the pill it’s bloating, gas, gas, just that, yes, it produces a lot of gas and bloating in the belly.” (Ines, Age 32, on PrEP three months, actively enrolled) Soledad, another participant nearing the end of the study, described that her side effects persisted for approximately five months and had only resolved in the month prior: “I’m about to reach 6 months [taking PrEP]. It’s caused me a lot of problems in my body, for example, it’s given me a lot, a lot of headaches and diarrhea. (Soledad, Age 31, on PrEP for six months, actively enrolled)

While in some cases, PrEP persistence and study retention were not impacted by physical side effects, in others, participants who experienced severe short-term or lingering long-term side effects ultimately withdrew. For example, Luciana, who used PrEP intermittently prior to withdrawal, described ongoing physical side effects that impacted her ability to work:

“I’ve had a lot of side effects, even now I get very tired, it makes me very sleepy, it interferes a lot with my sleep. For example it gives me a sleep disorder, I don’t sleep now like I slept before… There are days it makes me very hungry, and days it makes me not hungry at all, there were a few problems with colic, these disorders related to eating, but very few. What has impacted me the most has been the sleep disorder, the sleepiness.” (Luciana, Age 24, intermittent prep user for three months, withdrew from study).

When asked to comment on whether and why these long term, persistent side effects were common, the lead facilitator commented, “Maybe because of lifestyle… the majority of girls… presented with side effects that lasted a long time, that weren’t momentary, I believe that it could also be… perhaps because of lifestyle. Remember that the girls live alone, many of them eat only once per day, they are not eating multiple times per day, so this lifestyle is completely different.” (Tania, age 34, study facilitator).

### Social side effects

Participants described the impact of PrEP on daily lives beyond physical side effects. Most prominently reported was the theme of HIV-related stigma when others learned of their PrEP use. Participants described perceptions among friends, family, and romantic partners that PrEP is only used for HIV-treatment and anyone taking the medication is HIV-positive. One participant recounted: “Those who I’m close with know, but, I’ve realized that with clients, they are very suspicious and they think that I have HIV and that the pill is for HIV.” (Yessica, Age 27, on PrEP 6 months, active participant) Similarly, disclosure of PrEP use was considered to deter sexual and romantic partners. “I have a friend who was taking PrEP and she was intimate with a guy, and when he saw the bottle he was shocked. He said that she was ill, all of these things, that he knew about this [drug], that people take this because they are sick, and he left my friend’s room terrified.” (Maritza, age 29, on PrEP five months, actively enrolled).

Participants commonly highlighted confusion between PrEP and antiretroviral therapy, given that Tenofovir/Emtricitabine is also used to treat HIV, which caused tension when explaining PrEP to others and stigma that impacted their interpersonal relationships. “Yes, one time my friend found me and asked me, ‘What’s this?’, and I said ‘I’m taking this that they gave me at [the study clinic]’. She started to look on her phone on Youtube, something like that, and told me ‘This is for HIV, you have HIV.’” (Esmeralda, age 31, actively enrolled.)

One participant who had dropped out of the study described feeling embarrassed about taking PrEP and thought others would perceive her to be living with HIV. She described taking measures to conceal PrEP use from others: “I hide it in a box, because it made me embarrassed that someone would see it and think that I am sick.” (Paola, age 23, on PrEP one month, withdrew from study).

### Economic side effects

Participants also highlighted how loss of income, when physical side effects or social stigma prevented their ability to earn, was an important indirect effect of PrEP use. This was described within a greater context of economic precarity frequently experienced by trans women in Lima:

“I think it’s the culture, it’s the way we’ve been brought up, somewhat neglected in things that we would see as important in terms of health, because when they educate you to take good care of your body… the same precariousness, marginalization which the state, which society imposes on us, to barely survive, to survive day by day…most of us only seek to survive day by day.” (Renata, age 27, study facilitator).

Often, physical side effects rendered participants unwell and unable to work. “No, I don’t take it anymore, I said no to feeling like that anymore, plus I miss work.” (Paola, age 23, on PreEP one month, withdrew from study) Side effects that impacted sleep schedule or induced fatigue were particularly problematic, impacting the ability to wake up in time for shifts or to stay awake throughout working hours. “It made me really sleepy, really tired, so much so that there were days in which I couldn’t even go out to work because I was sleeping, and this affected my income, my work.” (Luciana, Age 24, intermittent PrEP user for three months, withdrew from study).

The social stigma of appearing sick or unwell was a marked concern for sex workers, who were worried about losing clients. A study peer facilitator commented on her interaction with study participants, saying: “You didn’t tell me that I was going to get headache, that I would vomit… and look friend, I work, and how am I going to take this if I’m going to feel this sick? No one will want me like this, they are going to think I have HIV.” (Carolina, Age 24, Facilitator).

### Impacts on PrEP acceptability, uptake, and continuation

These social, physical, and economic side effects often deterred trans women from initially enrolling in the study or continuing to take PrEP after enrollment. When asked about whether experiencing side effects impacted PrEP continuation, a peer facilitator commented, “Some yes, some no.” (Carolina, Age 24, Facilitator) Among evaluation participants, 6 (17.5%) had stopped taking PrEP, predominantly within the first month, and cited side effects as their primary reason for discontinuing. One participant stated:

I took it for two weeks… the few times within those two weeks which I took it, it shocked me badly, it gave me diarrhea… I stopped taking it, it’s been a week since I stopped taking it. I stopped it. Yes, I stopped it.” (Julieta, age 22, took PrEP for two weeks before discontinuing, withdrew from study).

Among evaluation participants, 8 (23.5%) decided not to enroll in the study or initiate PrEP and cited PrEP side effects as an influence on their decision. For Nayely, who declined to enroll, anticipated side effects were the primary barrier, particularly because she felt that given her current prevention practices, she felt she was already sufficiently protected from HIV. “Well, I told the doctor, ‘But why take it, if I know that I am healthy?’ I mean if a condom breaks with a guy who isn’t healthy, then yeah, this [pill] protects you. But I told him, ‘I know I’m OK…And this pill, what if it gives me side effects?’” (Nayely, age 28, Screened and Never Enrolled).

Although anticipated and experienced side effects impacted acceptability, many participants reported continuing despite these problems. Almost half (n = 16, = 47.1%) of participants were actively taking PrEP at the time of interview and described their willingness to do so despite initial or persistent side effects. In these cases, other motivations, such as social support and HIV prevention benefits, outweighed unpleasant side effects and encouraged PrEP continuation. One participant described the importance of health communication from study staff:

“The first day that I took it, I took it at night… and the next morning I woke up with an unbearable headache, with stress, with a migraine… I couldn’t stand the pain. Then I took it again and when I went to work, I felt so tired as if I had carried sacks, my body hurt that much. I didn’t want to take it anymore, so I stopped…Because I didn’t like the pill, I said no, because I didn’t give it any importance. The counselors explained to me why it’s necessary to take the pill, and then I decided to start taking it again… I am going to continue taking it, because I also have other friends who are taking this and it has helped them, they are fine.” (Raquel, age 25, on PrEP intermittently for two months, active participant).

Several participants commented on the benefits of PrEP for themselves, and for the trans community more broadly, including HIV prevention and feelings of security or empowerment. These participants noted the heightened frequency of HIV exposure faced by trans women, particularly those exposed through clients, and noted that PrEP provided an added layer of protection to mitigate this risk. Further, some participants commented that the intervention workshops provided a sense of community, social support, and peer-to-peer education which supported daily adherence.

## Discussion

Findings from this study highlight that PrEP side effects experienced by trans women in Peru are multidimensional and encompass physical, economic, and social impacts. These factors collectively impact the acceptability and persistence of PrEP use among trans women, potentially also affecting community-level PrEP uptake as others learn from their experiences [22, 30]. In some cases, long-term persistence, and the desire to continue PrEP were not due to an absence of side effects, but belief that the conferred benefits outweighed any negative experiences. This research underscores the importance of documenting and understanding the unique experiences of trans women who take PrEP, specifically regarding the complex side effects associated with PrEP and strategies to overcome them (including social support and education about the benefits of PrEP), to support uptake and adherence.

Physical side effects described by interview participants (nausea, diarrhea, fatigue, headache) were consistent with those described in the existing PrEP literature [24]. However, the severity and longevity of side effects, sometimes persisting up to 6 months, calls into question the validity of the concept of “startup syndrome”, traditionally lasting up to one month after initiating PrEP, for trans women in Peru and other LMIC settings [24]. In the larger TransPrEP study from which interview participants were recruited, adherence was generally poor, with protective levels of Tenofovir found in less than one-third of participants, and intermittent use described by some participants in this qualitative analysis [15]. It is possible that persistent symptoms are associated with incomplete adherence, or that participants were trapped in a cycle where experiencing side effects resulted in inconsistent use, and inconsistent use led to recurrent side effects. Prior analysis of the iPrEx-OLE (Open Label Extension) trial reported an association between lower adherence and ongoing gastrointestinal symptoms at the one-month mark but was unable to characterize whether gastrointestinal symptoms preceded non-adherence, or whether poor adherence and intermittent use caused recurrent gastrointestinal symptoms [24].

Results from the current study suggest that it is important to acknowledge and understand that among trans women, self-reported physical side effects may last longer, be more severe, and extend beyond the traditional one-month mark. Further, uncommon side effects (e.g., somnolence) may occur more frequently. Food insecurity and inadequate nutrition related to poverty were described by several participants, and these factors may plausibly alter how individuals experience gastrointestinal and other side effects, given the impact of meals on the bioavailability of Tenofovir [35, 36]. Contextual realities must be considered to, at the very least, understand how ‘start up syndrome’ may differ among trans women, and how it may have differential impacts depending on occupation (i.e., sex work), food, and housing stability. When side effects are dismissed as transient and short-term, it negates the very real experiences of trans women for whom this is not the case [30, 37, 38]. Previously published findings from this study have highlighted that minimizing questions or concerns about physical side effects (for example, patients’ questions about interactions between PrEP and gender affirming hormone therapy) may heighten mistrust of PrEP services [30]. Future research should examine the mechanisms impacting side effect longevity and severity among trans women, and on designing wrap-around strategies that may better support trans women taking PrEP. For example, this may include the role of nutritional intake (including food and water, and timing of meals in relation to PrEP dose) on the longevity of PrEP side effects.

Economic side effects of PrEP among trans women sampled include an inability to work, such as missing a shift due to physical side effects or grappling with extreme fatigue while working. Participants described factors such as a loss of sex work clients due to nausea or vomiting, fear that clients would perceive them to be ill, or having to pay out of pocket for over-the-counter medication to alleviate symptoms. Financial barriers to PrEP use have largely focused on the direct costs of paying for PrEP medication (i.e., paying for PrEP medication, paying for regular laboratory testing, lack of insurance coverage). Our findings advance existing literature by underscoring the importance of assessing indirect expenses (i.e., the cost of over-the-counter medications purchased to alleviate feelings of side effects), as well as the opportunity cost of missing work [22].

Participants also described social stigma related to taking a medication that is commonly used to treat HIV, such as friends or sexual partners seeing their pill bottle and assuming they were living with HIV, or researching medicines that comprise PrEP online. While some described attempting to educate friends or family about PrEP, others concealed their PrEP use and pill bottles to avoid questioning. This result is consistent with findings from other settings, where experienced or anticipated social stigma deters PrEP use [3]. New PrEP modalities, such as long-acting injectable cabotegravir, could potentially increase privacy and alleviate the stigma associated with carrying a pill bottle for some people [39, 40]. As these options become increasingly available worldwide, it is critical to continue evaluate the specific perceptions and experiences of trans women to understand the complexity of social side effects and potential impact of PrEP uptake and persistence by modality.

Conceptualizing social and economic experiences as “side effects”, unintended, and often detrimental, secondary effects that *are attributed to PrEP use* (for example, HIV stigma resulting from a family member finding a PrEP bottle), is crucial to advancing knowledge of the potential negative impacts of PrEP and how to best mitigate them. While many traditional “barriers” to PrEP use identified in the literature exist in the lives of trans women, such as reduced access to healthcare services, PrEP use may result in new, added stressors, that can layer onto existing vulnerabilities. For example, the cost of missing work when living below the poverty line, or the danger of social stigma related to HIV when already facing gender-based stigma [21]. It is vital to understand PrEP side effects and consider how these may exacerbate existing stressors, including but not limited to gender-related stigma and discrimination, especially in the Latin American context where levels of social exclusion, familial rejection, transphobic violence and transfemicide are extreme [31, 41].

The intersection of physical, social, and economic side effects was especially pronounced among trans women reporting sex work, who described harms including loss of income and damage to client relationships when feeling too unwell to work or when perceived to be living with HIV. Given that trans women sex workers are one of the priority populations for PrEP, it is crucial to understand the impact of PrEP on trans women’s’ ability to earn a living. Any interventions designed to support trans women through the startup syndrome and towards long-term adherence must be rooted in a thorough understanding of these and other experiences taking PrEP, and in the lived realities of trans women.

### Strengths and limitations

It is important to note the limitations of this research when interpreting findings. Our sample consists of adult trans women living in Lima who enrolled in a longitudinal PrEP study, and additional research is needed to understand the complexity of PrEP side effects and their impacts among trans women in other geographic areas of Peru and global settings. Further, the primary aim of the qualitative evaluation was to assess overall experiences in the study, and reasons for declining to enroll or to remain. While experiences taking PrEP, including side effects, were queried as part of the interview guide and emerged as an important salient theme, this question was not a primary aim of the interviews. Thus future work should focus on these experiences in greater depth. Finally, data presented in this manuscript were collected prior to the COVID-19 pandemic and should be interpreted within this context. While COVID-19 has had a lasting effect on Latin American health systems, and the global PrEP landscape has changed to include long-acting injectable regimens, PrEP is still not readily available in Peru beyond demonstration programs, and at the time of publication, uptake remains limited [11, 12, 14]. As public sector implementation of PrEP begins to scale up following the recent inclusion of oral PrEP in prevention guidelines for “high risk” populations, these findings are still relevant to inform the continued expansion and optimization of PrEP services for trans women [13].

Nevertheless, this study has several strengths. First, recruitment and implementation of interviews were conducted in partnership with trans community leaders. Further, study findings are unique in that our sample includes participants who were given the opportunity to take PrEP at no cost, but declined, as well as women who enrolled in a PrEP trial and used PrEP for varying lengths of time, some opting to continue. In contrast, others discontinued after initially trying PrEP. Many studies of PrEP barriers and acceptability are based on samples of PrEP-naïve participants who describe their feelings or knowledge about PrEP without having the opportunity to take it.

## Conclusion

Understanding the unique experiences of trans women taking PrEP is crucial to informing tailored interventions to improve PrEP uptake and persistence. This research highlights that physical, economic, and social side effects of PrEP impact acceptability among trans women who have initiated PrEP. Further, multidimensional side effects may amplify and exacerbate existing stressors, including stigma, violence, transphobia, and economic exclusion. A nuanced understanding of the contextual realities in which PrEP is delivered across varied global settings is critical to supporting trans women initiating PrEP, and ultimately improving HIV prevention strategies.

### Electronic supplementary material

Below is the link to the electronic supplementary material.

Supplementary Material 1

## Data Availability

The data that support the findings of this study are available upon reasonable request sent to the Principal Investigator, Dr. Jesse L. Clark (JLClark@mednet.ucla.edu). The data are not publicly available due to privacy or ethical restrictions.
