# Supplementary material for: Social, economic, and physical side effects impact PrEP uptake and persistence among transgender women in Peru
Source: BMC Public Health. 2024 Jul 25;24:1985. doi: 10.1186/s12889-024-19474-x (PMC11270771; doi:10.1186/s12889-024-19474-x)
Supplement: Supplementary file 1 — Supplementary Material 1 [file 12889_2024_19474_MOESM1_ESM.pdf]

Additional File 1: Additional File 1, Interview Guide (pdf)

TRANSPREP FINAL EVALUATION INTERVIEW

Introduction

Hello, my name is (Interviewer). Thank you for coming to meet with me today. I appreciate your willingness to share your thoughts and feelings about your participation in the TransPrEP study. As you know, this interview is part of a research project to learn about how to best offer PrEP to transgender women. Do you have any questions before we begin?

Review of Consent Form

I'm going to pause here and ask you to read through the informed consent. [PAUSE]

There are a few things in the consent form that are especially important, so I want to go over those with you now.

If at any time you feel uncomfortable about any of the questions, you do not have to answer. If you need to take a break at any point, let me know. Also, if at any time you want to end the interview that is fine. Just let me know.

Do you want to ask any questions before we go on?

[ASK THE PARTICIPANT TO SIGN THE CONSENT FORM].

Interview [TURN ON THE RECORDER].

INTRODUCTION:

For the recorder, "Hello my name is [Interviewer Name]. Today is [Date, Time] and we are interviewing participant number [ID Number] at [Location]. For the record, may I have your permission to audio record this conversation?"

PART 1: DEMOGRAPHICS

Great. Before we begin, would you mind answering a few brief questions about yourself?

1. *What is your age?*

2. *What do you do for a living?*

3. *Are you originally from Lima?*

- *Probe: If no, how long have you lived here?*
- *Probe: Where are you originally from?*

PART 2: GENERAL RESEARCH HISTORY

As you know, we are here to talk about the "TransPrep" study. TransPrep is a medical research study.

1. *Other than TransPrEP, have you ever participated in other medical research studies in the past?*

2. *If so, do you remember the name of the study? Do you remember what this study was about?*
   - *Probe: In this study, did you receive mediation of any kind?*

3. *How do you feel about personally participating in medical research?*

4. *As a trans woman, how do you feel about research projects that focus specifically on recruiting transgender women to participate?*

5. *Among trans women in Lima, what is the general perspective about participating in research projects?*

PART 3: GENERAL TRANSPREP EXPERIENCE

1. *Ok, now I'd like to ask you specifically about the TransPrep study. How did you initially hear about the TransPrep study?*

2. *When you first learned about TransPrep, what initially interested you about the study?*

3. *What motivated you to participate in the study?*

4. *When you learned about the study, what was your understanding of what would take place?*

5. *How does your experience compare with what you expected of the study?*

6. *Starting from the beginning, can you tell me a little about your history with Transprep? Can you describe the types of study activities that you participated in?*

7. *How would you describe your overall experience as a participant in TransPrEP?*
   - *PROBE: Can you provide one or two specific examples that illustrate your experience?*

8. *Can you tell me about the things you liked most about the TransPrep study?*

9. *Can you tell me about the things that you did not like about the TransPrep study?*

10. [**FOR DROPOUTS/NON-ENROLLEES**] *Can you tell me why you chose to stop participating in the TransPrep study?(If about PrEP, probe on what aspects about PrEP they didn't like)*

11. *Was there anything we could have done to have encouraged you to stay in the study?*

PART 4: PREP

1. *Now thinking about different aspects of the study, I'd like to ask you a little bit about the medication PrEP. I know it's a little confusing, but as a reminder, "TRANSPREP" is the name of the research study. "PREP" is the name of a medication. Can you tell me what you know about the medication PrEP?*

2. *Did you begin taking PrEP as a part of this study?*
   - *Probe: If yes – are you still taking PrEP, or did you ever decide to stop?*
   - *Probe: You mentioned that you stopped taking PrEP; why did you decide to stop taking PrEP?*
   - *Probe: You mentioned that you never started taking PrEP. Can you tell me why you chose not to take PrEP?*

3. *If you had any questions regarding PrEP, where they addressed by study staff?*

4. *Do you have any concerns about PrEP?*

5. *[SKIP if they never started PREP] How was your experience with taking PrEP?*

6. *[SKIP if they never started PREP] Did you have any side-effects? If so, did you feel you received adequate medical attention?*

7. *[SKIP if they never started PREP] Do you think that you will continue to take PrEP even after the study has finished?*
   - *PROBE: If so, how do you plan to access PrEP?*

8. *Do you think PrEP is a good HIV prevention tool for the TW community?*

9. *Would you recommend PrEP to a trans woman friend? Why or why not?*

10. *Would you recommend PrEP to a romantic partner? Why or why not?*

11. *Do you think PrEP is a good HIV prevention tool for you, specifically?*

PART 5: CLINICAL EXPERIENCE

I'd like to ask you a little bit about your experience attending the [REDACTED] clinic as a part of this study.

1. *How was your experience with study physicians?*
   - *PROBE: how do you feel you were treated by physicians?*

2. *How was your experience with study counselors?*
   - *PROBE: how do you feel you were treated by counselors?*

3. *How was your experience with general staff? (For example, front desk staff, pharmacists)*

PART 6: GROUP WORKSHOPS

1. *Part of the Transprep study involved participating in group workshops with other trans women at the Feminas house. Did you participate in any of these workshops?*

2. *How was your experience with the TransPrEP workshops?*

3. *How did you feel about discussing sensitive topics like HIV and sexual health in a group with other trans women?*

4. *Did you know any of your group mates prior to beginning the study?*

5. *Would you consider maintaining contact with your group mates post-study end? Why or why not?*

6. *Do you have any suggestions for improving how the TransPrEP workshops were run?*

7. *What was your experience working with peer facilitators during the study?*

8. *Do you have any suggestions for improving how the care and information provided by peer facilitators?*

PART 7: STUDY LOGISTICS

1. *I'd like to talk a little bit about the time commitment involved with the study. Between clinical visits, surveys, and group workshops, how did you feel about the amount of time you were asked to give?*

2. *How did the group workshops fit into your schedule? Did you find they were easy/difficult to make time for?*

3. *How did you feel about the level of organization and reliability of the group workshop schedule?*

4. *Can you describe what it was like to schedule and attend clinical visits at [redacted]?*

5. *How do you feel about the level of compensation that was offered for your participation in this study?*

PART 8: CONCLUDING QUESTIONS

1. *Based on your experience with TransPrEP, how would you feel about participating in another research study?*

2. *Prior to concluding we will be doing one more activity. Please turn your attention to the 10 cards in front of you. I will describe each image and will be asking you order them based on how problematic each aspect was (least liked on top to most liked on the bottom).*
   - *PROBE: Tell me more about the least liked aspects?*

3. *Is there anything else that you'd like to tell me?*

4. *Do you have any questions for me?*

[TURN OFF RECORDER].
